# Supplementary material for: Cocaine chemogenetics blunts drug-seeking by synthetic physiology
Source: Nature. 2025 Aug 27;646(8085):746–53. doi: 10.1038/s41586-025-09427-8 (PMC12527922; doi:10.1038/s41586-025-09427-8)
Supplement: Supplementary file 1 — Reporting Summary [file 41586_2025_9427_MOESM1_ESM.pdf]

nature portfolio

Corresponding author(s): Scott Sternson & Michael Michaelides

Last updated by author(s): 6/17/2025

Reporting Summary

Nature Portfolio wishes to improve the reproducibility of the work that we publish. This form provides structure for consistency and transparency in reporting. For further information on Nature Portfolio policies, see our Editorial Policies and the Editorial Policy Checklist.

Statistics

For all statistical analyses, confirm that the following items are present in the figure legend, table legend, main text, or Methods section.

n/a	Confirmed	
☐	☒	The exact sample size (*n*) for each experimental group/condition, given as a discrete number and unit of measurement
☐	☒	A statement on whether measurements were taken from distinct samples or whether the same sample was measured repeatedly
☐	☒	The statistical test(s) used AND whether they are one- or two-sided<br>*Only common tests should be described solely by name; describe more complex techniques in the Methods section.*
☐	☒	A description of all covariates tested
☐	☒	A description of any assumptions or corrections, such as tests of normality and adjustment for multiple comparisons
☐	☒	A full description of the statistical parameters including central tendency (e.g. means) or other basic estimates (e.g. regression coefficient) AND variation (e.g. standard deviation) or associated estimates of uncertainty (e.g. confidence intervals)
☐	☒	For null hypothesis testing, the test statistic (e.g. *F*, *t*, *r*) with confidence intervals, effect sizes, degrees of freedom and *P* value noted<br>*Give P values as exact values whenever suitable.*
☒	☐	For Bayesian analysis, information on the choice of priors and Markov chain Monte Carlo settings
☒	☐	For hierarchical and complex designs, identification of the appropriate level for tests and full reporting of outcomes
☒	☐	Estimates of effect sizes (e.g. Cohen's *d*, Pearson's *r*), indicating how they were calculated

Our web collection on statistics for biologists contains articles on many of the points above.

Software and code

Policy information about availability of computer code

Data collection	MED-PC v.4.2 (Med Associates, Self-administration), Tucker Davis Technologies Synapse Software v. 95-44132P (Photometry), Nucline NanoScan 3.04.025 (Mediso, PET), LAS X 3.4 (Leica, Immunohistochemistry), pClamp 11.
Data analysis	Microsoft Excel 2024 and 2025, Graphpad Prism 10, MatlabR2016 & MatlabR2023b, Statistical Parametric Mapping (SPM12), PMOD v4.1, Sigma Plot 11.0

For manuscripts utilizing custom algorithms or software that are central to the research but not yet described in published literature, software must be made available to editors and reviewers. We strongly encourage code deposition in a community repository (e.g. GitHub). See the Nature Portfolio guidelines for submitting code & software for further information.

Data

Policy information about availability of data

All manuscripts must include a data availability statement. This statement should provide the following information, where applicable:

- Accession codes, unique identifiers, or web links for publicly available datasets
- A description of any restrictions on data availability
- For clinical datasets or third party data, please ensure that the statement adheres to our policy

All data supporting the in vivo findings are available within this article and in the extended data. DNA constructs are available from Addgene.com under Materials Transfer Agreement. Data for model of cocaine bound to AChBP is available at PDB: 2pgz.

Research involving human participants, their data, or biological material

Policy information about studies with human participants or human data. See also policy information about sex, gender (identity/presentation), and sexual orientation and race, ethnicity and racism.

Reporting on sex and gender	N/A
Reporting on race, ethnicity, or other socially relevant groupings	N/A
Population characteristics	N/A
Recruitment	N/A
Ethics oversight	N/A

Note that full information on the approval of the study protocol must also be provided in the manuscript.

Field-specific reporting

Please select the one below that is the best fit for your research. If you are not sure, read the appropriate sections before making your selection.

☒ Life sciences ☐ Behavioural & social sciences ☐ Ecological, evolutionary & environmental sciences

For a reference copy of the document with all sections, see nature.com/documents/nr-reporting-summary-flat.pdf

Life sciences study design

All studies must disclose on these points even when the disclosure is negative.

Sample size	Sample sizes were estimated using G*Power or based on experience from past work.
Data exclusions	Rats were excluded if they lost catheter patency or due to lack of AAV transduction. Self-administration data were excluded for sessions where the infusion line disconnected from the rat's catheter port.
Replication	Detailed methods are provided to aid in replication by others. Behavioral, imaging and photometry experiments were replicated in at least 2 or more cohorts and by more than one experimenter. In vitro assays (binding, ELISA) were performed with at least two replicates and all replicate attempts were successful.
Randomization	Rats were randomly assigned to experimental groups and treatment conditions. Samples for in vitro assays (e.g. binding, ELISA) were also randomized with respect to treatment conditions.
Blinding	Experimenters were not blinded to group allocation during data collection for behavioral, imaging, and photometry experiments. For behavioral and photometry experiments data were collected by one researcher and analyzed by another. Data were analyzed blind if applicable but experimenters were always aware of the conditions. Immunohistochemistry experiments were performed blinded.

Reporting for specific materials, systems and methods

We require information from authors about some types of materials, experimental systems and methods used in many studies. Here, indicate whether each material, system or method listed is relevant to your study. If you are not sure if a list item applies to your research, read the appropriate section before selecting a response.

Materials & experimental systems

n/a	Involved in the study	
☐	☒	Antibodies
☐	☒	Eukaryotic cell lines
☒	☐	Palaeontology and archaeology
☐	☒	Animals and other organisms
☒	☐	Clinical data
☒	☐	Dual use research of concern
☒	☐	Plants

Methods

n/a	Involved in the study	
☒	☐	ChIP-seq
☒	☐	Flow cytometry
☒	☐	MRI-based neuroimaging

Antibodies

Antibodies used	rat monoclonal anti-mCherry (Clone 16D7, Invitrogen M11217), chicken polyclonal anti-GFP (Abcam ab13970), goat-anti rat immunoglobulin G (IgG) (H+L) crossabsorbed, Alexa Fluor 594 (Invitrogen, A11007), goat-anti chicken immunoglobulin Y (IgY) H&L (Alexa Fluor 488) (Abcam ab150173)
Validation	Primary antibodies were validated for immunohistochemistry by the specific vendor and confirmed in our lab where they showed selective immunolabeling in AAV targeted sites using AAVs with established fluorescent reporters.<br><br>rat monoclonal anti-mCherry (Clone 16D7, Invitrogen M11217):<br>https://www.thermofisher.com/antibody/product/mCherry-Antibody-clone-16D7-Monoclonal/M11217<br><br>chicken polyclonal anti-GFP (Abcam ab13970):<br>https://www.abcam.com/en-us/products/primary-antibodies/gfp-antibody-ab13970<br><br>goat-anti rat immunoglobulin G (IgG) (H+L) crossabsorbed, Alexa Fluor 594 (Invitrogen, A11007):<br>https://www.thermofisher.com/antibody/product/Goat-anti-Rat-IgG-H-L-Cross-Adsorbed-Secondary-Antibody-Polyclonal/A-11077<br><br>goat-anti chicken immunoglobulin Y (IgY) H&L (Alexa Fluor 488) (Abcam ab150173):<br>https://www.abcam.com/en-us/products/secondary-antibodies/goat-chicken-igy-h-l-alexa-fluor-488-ab150169

Eukaryotic cell lines

Policy information about cell lines and Sex and Gender in Research

Cell line source(s)	ATCC CRL-1573 cells (HEK-293, passages 40-49)
Authentication	Cell lines were not authenticated after receipt from ATCC
Mycoplasma contamination	Cell lines were tested for mycoplasma contamination. If contaminated cultures were detected, then they were not used for experiments.
Commonly misidentified lines<br>(See ICLAC register)	HEK cells can be misidentified as HeLa cells, but our lab received CRL-1573 HEK 293 cells directly from ATCC. We do not have HeLa cells in our lab.

Animals and other research organisms

Policy information about studies involving animals; ARRIVE guidelines recommended for reporting animal research, and Sex and Gender in Research

Laboratory animals	Adult (8-10 weeks) Long Evans (CrL:Le) #006 rats, TH-cre rats, adult (8-10 weeks) Sprague Dawley (Crl:SD) #400 rats, C57Bl/6J mice (male, 4-8 weeks)
Wild animals	The study did not involve wild animals
Reporting on sex	We used male rats for all experiments unless specified in the methods.
Field-collected samples	The study did not involve animals collected from the field
Ethics oversight	Experiments and procedures complied with ethical regulations for animal testing and research, followed the NIH guidelines and were approved by the NIDA animal care and use committee. Experiments and procedures complied with ethical regulations for animal testing and research and were approved by the Janelia Research Campus and UCSD animal care and use committees.

Note that full information on the approval of the study protocol must also be provided in the manuscript.

Plants

Seed stocks	*Report on the source of all seed stocks or other plant material used. If applicable, state the seed stock centre and catalogue number. If plant specimens were collected from the field, describe the collection location, date and sampling procedures.*
Novel plant genotypes	*Describe the methods by which all novel plant genotypes were produced. This includes those generated by transgenic approaches, gene editing, chemical/radiation-based mutagenesis and hybridization. For transgenic lines, describe the transformation method, the number of independent lines analyzed and the generation upon which experiments were performed. For gene-edited lines, describe the editor used, the endogenous sequence targeted for editing, the targeting guide RNA sequence (if applicable) and how the editor was applied.*
Authentication	*Describe any authentication procedures for each seed stock used or novel genotype generated. Describe any experiments used to assess the effect of a mutation and, where applicable, how potential secondary effects (e.g. second site T-DNA insertions, mosiacism, off-target gene editing) were examined.*
